# Safe and Effective Histotripsy Ablation of Human Liver Tumors in a Genetically Modified Porcine Model

**DOI:** 10.3390/cancers18152432

**Published:** 2026-07-29

**Authors:** Tamalika Paul, Jessica Gannon, Manali Powar, Cora Youngs, Cassandra S. Poole, Carley M. Elliott, Mackenzie K. Woolls, Khan Imran Mohammad, Sherrie Clark-Deener, Christopher Byron, Michael Edwards, Sheryl Coutermarsh-Ott, Kristin Eden, Kiho Lee, Timothy J. Ziemlewicz, Eli Vlaisavljevich, Irving C. Allen

**Affiliations:** 1 Department of Biomedical Sciences and Pathobiology, Virginia-Maryland College of Veterinary Medicine, Blacksburg, VA 24061, USA; tamalika@vt.edu (T.P.); cassandrapoole@vt.edu (C.S.P.); slc2003@vt.edu (S.C.-O.); keden@vt.edu (K.E.); 2 Department of Biomedical Engineering and Mechanics, Virginia Polytechnic Institute and State University, Blacksburg, VA 24061, USA; jess98@vt.edu; 3 Graduate Program in Translational Biology, Medicine, and Health, Virginia Tech, Roanoke, VA 24016, USA; manali21@vt.edu (M.P.); carleye6@vt.edu (C.M.E.); mwoolls@vt.edu (M.K.W.); imrankhan@vt.edu (K.I.M.); 4 College of Science, Virginia Tech, Blacksburg, VA 24061, USA; corayoungs16@gmail.com; 5 Department of Large Animal Clinical Sciences, Virginia-Maryland College of Veterinary Medicine, Blacksburg, VA 24061, USA; sherrie@vt.edu (S.C.-D.); cbyron@vt.edu (C.B.); 6 Department of Small Animal Clinical Sciences, Virginia-Maryland College of Veterinary Medicine, Blacksburg, VA 24061, USA; mredward@vt.edu; 7 Department of Basic Science Education, Virginia Tech Carilion School of Medicine, Roanoke, VA 24016, USA; 8 Division of Animal Science, College of Agriculture Food and Natural Resources, University of Missouri, Columbia, MO 65211, USA; kiholee@missouri.edu; 9 Department of Radiology, University of Wisconsin School of Medicine, Madison, WI 53705, USA; tziemlewicz@uwhealth.org

**Keywords:** pig, focused ultrasound, cancer, hepatocellular carcinoma, pancreatic adenocarcinoma

## Abstract

Background: Liver cancers are a major cause of morbidity and mortality in patients where effective, non-invasive treatment options remain limited. Objective: Histotripsy is a non-invasive, non-thermal, image-guided focused ultrasound method of ablation that mechanically disrupts cells and offers a range of potential advantages over other ablation modalities. The lack of physiologically and anatomically relevant animal models of human liver cancer has significantly hindered biomedical device development, including histotripsy. Methods: To address these limitations, we developed a clinically relevant large animal orthotopic, dual-tumor model of human liver cancer and utilized these unique animals to evaluate the safety and efficacy of histotripsy. Here, we utilized immunocompromised pigs with genetic modifications in their IL-2RG and RAG2 genes and orthotopically engrafted human hepatocellular carcinoma (HepG2/C3A) and pancreatic adenocarcinoma (Panc-1) cells within the liver. The models were designed to recapitulate primary and metastatic liver tumor phenotypes. Results: Histotripsy enabled real-time visualization of the treatment by the formation of bubble clouds and accurate targeting of the lesions. Histological analysis confirmed the engraftment of tumor cells and the ablation of targeted tissue. Serum biomarkers demonstrated no significant differences in bilirubin, ALT, ALKP, or CK post-treatment, suggesting that histotripsy treatment was well tolerated with minimal hepatic dysfunction or hepatocellular injury. Conclusions: These findings establish a novel, clinically relevant porcine model of primary and metastatic liver tumors and demonstrate the safety and feasibility of using this model for evaluating histotripsy as a noninvasive modality for precise tumor ablation.

## Simple Summary

Liver cancer remains a major cause of death worldwide, yet treatment options that can destroy the tumors without surgery remain limited. Histotripsy is a non-invasive ultrasound technology that uses focused ultrasound waves to mechanically break down tumors without the use of heat and has shown promise as a precise method for tumor ablation. However, the development and evaluation of this technology have been limited by the lack of animal models that closely replicate human liver cancer. In this study, we established a clinically relevant pig model of both primary and metastatic liver tumors and used it to access the safety and effectiveness of histotripsy. The treatment successfully targeted and destroyed tumor tissue while preserving overall liver function. These findings provide an important new platform for testing liver cancer therapies and support the continued development of histotripsy as a safe, non-invasive approach for treating liver tumors.

## 1. Introduction

Hepatocellular carcinoma is a significant global health burden as a primary liver malignancy [1,2,3,4]. The liver is also the most common site of pancreatic ductal adenocarcinoma (PDAC) metastasis and represents the most clinically consequential location of disease progression [5,6]. The presence of hepatic metastasis is a major driver of mortality in PDAC, which remains one of the deadliest malignancies due to its aggressive nature, early dissemination and limited therapeutic options [1,7,8]. Most patients with advanced pancreatic cancer develop liver metastases during the course of their disease, making the control of hepatic tumor burden critical for improving patient outcomes [1,4,8]. The liver is not simply a passive recipient of metastatic cells; its highly vascular architecture, structure, and inherently tumorigenic immune environment create conditions that favor tumor implantation and immune evasion [4,9,10]. Despite advances in systemic chemotherapy, durable control of liver metastasis remains rare, highlighting the need for improved locoregional treatment strategies to address metastatic disease within the liver [2].

Although the synchronous occurrence of PDAC and HCC in the same patient is less common than either alone [11,12,13], the liver frequently contains heterogeneous tumor burdens, whether due to the multifocal primary disease or metastatic spread [9,14,15,16]. Clinically, physicians must often manage complex hepatic tumor landscapes that differ in origin, biology, and therapeutic response. However, most clinical models examine either metastatic pancreatic cancer or primary liver cancer in isolation, failing to reflect this biological complexity. In addition, genetically engineered porcine cancer models, such as the Oncopig Cancer Model, have been developed to investigate tumorigenesis in porcine tissues by activation of tissue-specific induction of tumors in immunocompetent pigs through tissue-specific activation of oncogenic mutations [17]. While these models provide valuable insights into cancer progression, to better model the hepatic tumor environment, we developed an orthotopic, dual-tumor, porcine liver model in which human pancreatic cancer and human hepatocellular carcinoma coexist within the same liver. In the present study, we used our RAG2/IL2RG immunocompromised pig model [18,19,20,21] and engrafted human Panc-1 pancreatic cancer cells and human HepG2/C3A hepatocellular carcinoma cells into anatomically challenging regions of the liver, mimicking sites often considered difficult to access or clinically treat. By introducing controlled heterogeneity into a single organ, this platform enables the study of tumor microenvironment interactions, differential growth patterns, and treatment responses in a setting that more closely resembles the clinical challenges encountered in hepatic oncology.

Locoregional ablation techniques, such as radiofrequency [22], microwave [23], cryoablation, and IRE [24,25], are often deployed to treat liver tumors [26,27,28,29]. While effective in selected settings, all these modalities require invasive surgical procedures, and most of these approaches rely on thermal injury. Thermal approaches can be limited by heat-sink effects near large vessels, which is a phenomenon that occurs during the treatment when blood flow in nearby vessels carries heat away from the ablation zone. This effect reduces the temperature achieved in the tumor tissue, resulting in imprecise ablation margins and collateral damage to surrounding liver tissue [30]. In highly vascular organs, such as the liver, these limitations may reduce treatment uniformity and leave viable tumor cells at the periphery of the ablation zones. Furthermore, thermal destruction does not necessarily promote favorable immune activation [31].

Histotripsy is a non-thermal, non-invasive, and non-ionizing focused ultrasound tissue ablation method that creates sharply demarcated ablation zones while protecting surrounding structures [32,33,34,35,36,37]. Conventional High Intensity Focused Ultrasound (HIFU) primarily relies on thermal energy to induce necrosis, whereas histotripsy uses high-amplitude, short-duration ultrasound pulses to generate acoustic cavitation that mechanically fractionates tissue. These characteristics are particularly advantageous for solid tumors, especially pancreatic tumors or liver tumors, which are frequently located at difficult locations like next to a blood vessel, the bile duct, and the duodenum, where precise tissue destruction while preserving surrounding anatomy is essential [32,33,38].

Emerging pre-clinical data suggest that histotripsy not only mechanically destroys tumors but also releases antigens and immune-stimulating damage-associated molecular patterns, potentially enhancing anti-tumor immune responses [39]. Its recent clinical trial evaluation for liver tumors, including primary and metastatic disease, demonstrates feasibility, safety, and precise tissue targeting [39,40]. The data thus far are highly supportive of the potential for histotripsy to be deployed as a next-generation tumor ablation modality. However, its performance in heterogeneous hepatic environments, especially with tumors of distinct biological origins, remains to be fully explored [18,37,41,42,43,44].

In the present study, we introduce a dual-tumor porcine liver model combined with histotripsy to demonstrate the feasibility and potential advantages of precision ablation in complex hepatic disease. This study highlights the model’s use and relevance for hepatic oncology research and clinical translation. Following successful tumor establishment, tumors were treated using histotripsy. Clinically relevant treatment parameters were systematically evaluated, and procedural feasibility, ablation, precision, and technical limitations were defined in this large-animal model. Importantly, the anatomical scale and hepatic architecture of the model provide a translationally relevant framework from which treatment parameters and procedural considerations may be extrapolated to human applications. Collectively, this work builds on prior studies of histotripsy using the immunocompromised porcine tumor engraftment model and extends the application of histotripsy to a complex hepatic setting containing two distinct human malignancies.

## 2. Materials and Methods

### 2.1. Generation of Immunocompromised Pigs by RAG2/IL2RG Deletion

RAG2/IL2RG double-knockout pigs were generated using a CRISPR/Cas9-mediated genome editing approach as previously reported in the literature [18,19,20,21,45]. Piglets were delivered by sterile hysterectomy and immediately transferred under aseptic conditions into isolators to maintain germ-free housing. All procedures were conducted in accordance with the National Institutes of Health (NIH) guide for the Care and Use of Laboratory Animals and were approved by the Virginia Tech Institutional Animal Care and Use Committee (IACUC). The immunocompromised status of the pigs was confirmed by genotyping RAG2 and IL2RG loci using PCR followed by Sanger sequencing, which verified the mutations in the RAG2/IL2RG genes [18,19,20,21].

### 2.2. Surgical Implantation of HepG2/C3A and Panc-1 Cells into the Pig Liver

The HepG2/C3A cells (ATCC, CRL-10741) were maintained in Eagle’s minimum essential medium (MEM; Gibco-Thermo Fisher, Waltham, MA, USA) supplemented with 10% FBS, 2 mM L-glutamine, 100 IU/mL antibacterial-antimycotic (Gibco-Thermo Fisher, Waltham, MA, USA), and 1% minimal essential amino acids. HepG2/C3A cells were grown on rat collagen-coated culture plates (Gibco-Thermo Fisher, Waltham, MA, USA). Human pancreatic cancer cells (Panc-1) were cultured in RPMI medium supplemented with 10% FBS, 1% Normocin and, 1% non-essential amino acids. To generate well-defined tumors, the cells were resuspended in Matrigel at a concentration of 6 × 10^6^ cells per 100 μL and were maintained on ice until they were orthotopically injected into the pigs [18,19]. Following anesthetic and surgical protocols previously described [18,19] Panc-1 cells and HepG2/C3A cells were engrafted into the medial lobes of the liver. HepG2/C3A cells were injected into the right medial lobe, and Panc-1 cells were injected into the left medial lobe. Tumors were allowed to develop for 2 weeks prior to histotripsy treatment, reaching approximately 0.5–1 cm in diameter.

### 2.3. CT Imaging Prior to Histotripsy Treatment

**Figure 1:**
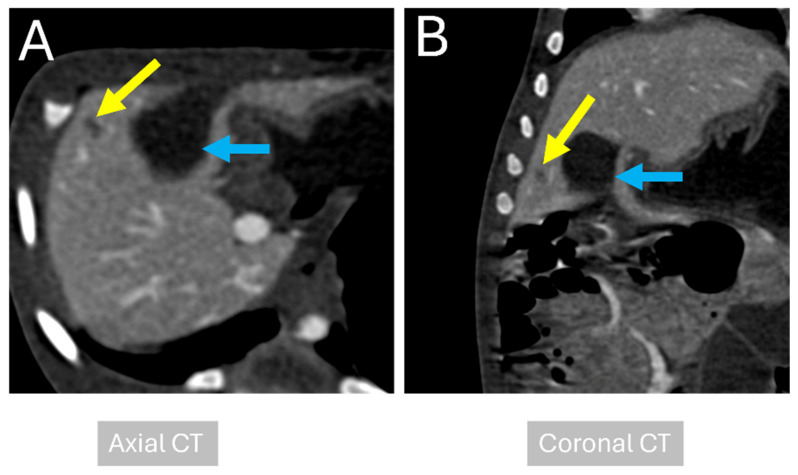
Successful tumor engraftment and CT visualization. (**A**) Representative axial and (**B**) coronal pre-Tx CT image, indicating successful engraftment of tumor next to the gall bladder. Yellow arrows show site of successfully engrafted tumor. Blue arrow is gallbladder for location reference. Pre-ablation Hounsfield units: Pre-contrast: 75.25 HU, post-contrast: 85 HU.

To confirm tumor establishment and assess the treatment response, contrast-enhanced computed tomography (CT) imaging was performed [18,42,43]. In the first round, one pig underwent abdominal helical CT scanning immediately before the histotripsy treatment. Imaging included non-contrast scans followed by contrast-enhanced triple-phase scans (Figure 1). Animals were positioned supine and maintained under general anesthesia throughout the procedure. Imaging parameters included a tube rotation time of 0.5 s, slice thickness of 1 mm, and a pitch factor of 0.828. Scans extended from cranial aspect of the diaphragm to the pelvic inlet. For contrast-enhanced imaging, iohexol (300 mg iodine/mL; Omnipaque 300, GE Healthcare, Chicago, IL, USA) was administered at a dose of 0.86 mL/kg using a power injector (Medrad Stellant, Indianola, PA, USA). Scan delay was triggered off the abdominal aorta at the level of the diaphragm for the arterial phase, measuring the mean of Hounsfield units (HU) in the non-enhanced aorta and adding 30 HU to determine the HU to trigger the system to begin scanning. The equilibrium phase was scanned after 3 min from the time the contrast medium injection started. The engrafted tumors exhibited poorly defined mild heterogeneous hypoattenuation compared to normal liver parenchyma (tumor: 63 HU, liver: 75 HU), with mild heterogeneous enhancement during the arterial phase (tumor: 95 HU, liver: 97 HU). Following the arterial phase, no further contrast enhancement occurred, (portal-phase tumor: 84–95 HU, liver: 104–113 HU), with retained mild heterogeneous contrast enhancement on the full delay phase (tumor: 94 HU, liver: 113 HU). CT images were independently reviewed by a veterinary radiologist (M.E.).

### 2.4. In Vivo Histotripsy Treatment

All animals were sedated with 2–4 mL/kg of Telazol-Ketamine-Xylazine (TKX) and maintained under general anesthesia (isoflurane) throughout all the procedures. At the time of treatment, all pigs weighed between 4 and 6 kg. As this study represented the first attempt to establish orthotopic dual liver and pancreatic tumor implantation in this model, a necropsy was performed on one untreated, tumor-implanted pig to confirm successful engraftment of tumor cells. Upon confirmation of tumor establishment, histotripsy treatment was initiated in subsequent animals [18].

Histotripsy was performed *in vivo* by targeting each tumor using a custom 32-element 500 kHz therapy transducer [18,42,43]. The transducer was driven by a custom high-voltage field-programmable gate array (FPGA) board (Altera DE0-Nano, Terasic Technology, Hsinchu, Taiwan) coupled to a pulser to generate short, ≤2 cycle histotripsy pulses. The transducer had a focal depth of 78 mm, with transverse and elevated aperture dimensions of 128 mm and 112 mm, respectively. Detailed system specifications have been previously reported [18]. The therapy transducer was mounted on a robotic micro positioner and integrated into a histotripsy treatment platform (HistoSonics, Inc., Plymouth, MN, USA). A 3 MHz curvilinear imaging probe (Model C52, Analogic Corp., Peabody, MA, USA) was coaxially aligned with the therapy transducer to provide real-time ultrasound guidance during the treatment (Figure S2). Treatment parameters were standardized across animals and delivered at a peak negative pressure of 25.51 ± 0.064 MPA (mean ± SD). In one treatment case, the applied pressure was varied during the treatment and ranged from 17.2 to 29.1 MPa approximately, a pulse repetition frequency of 500 Hz, and 1000 pulses per focal point. Planned 5 mm treatment margins surrounding the tumor boundary were incorporated to ensure coverage of the lesion and the immediate peritumoral tissue. Among the tumor-bearing animals, histotripsy treatment was performed in five pigs across two experimental rounds. In the first round, two pigs underwent histotripsy treatment, while in the second round, three pigs received histotripsy treatment, and in total, three animals underwent ultrasound imaging.

**Figure 2:**
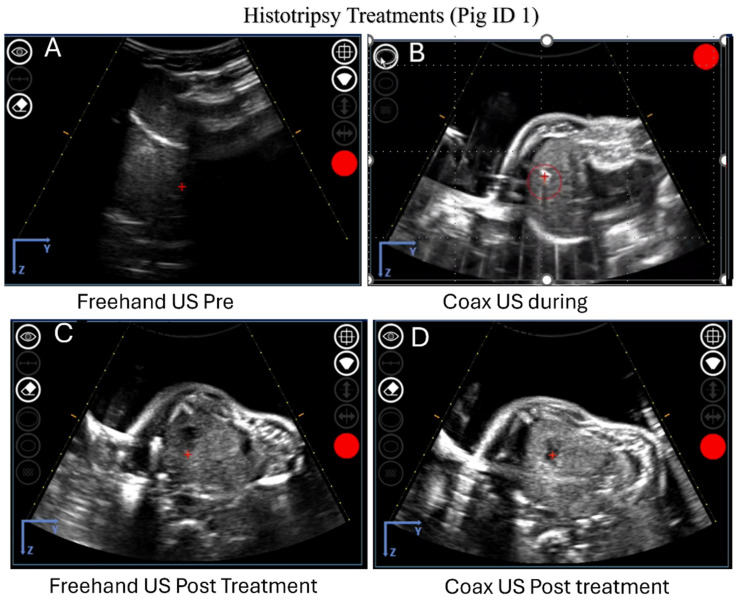
Ultrasound visualization of histotripsy treatment and immediate post-histotripsy effects in the pig liver model. (**A**) The liver was identified using freehand ultrasound imaging (red plus sign). (**B**) The liver remained visible on coaxial ultrasound imaging during treatment, and the histotripsy-induced bubble cloud was clearly observed (red plus sign). Bubble clouds were consistently visualized and maintained throughout treatment. Freehand ultrasound post-treatment indicates clear ablation zone. (**C**,**D**) Post-treatment freehand ultrasound imaging revealed a hypoechoic region corresponding to the treated area (red plus), which was observed immediately following histotripsy treatment.

Freehand ultrasound (imaging probe Model C5–2, Analogic Corp.) was used to provide real-time visualization of tumor location and procedural planning without the delivery of therapeutic pulses (Figure 2). The ultrasound was also used to identify the optimal acoustic window on the abdominal surface for treatment (Figure 2). Histotripsy was delivered using single-cycle pulses at a pulse repetition frequency (PRF) of 500 Hz with an automated volumetric spherical ablation strategy. Each tumor volume was scanned twice, delivering approximately 1000 pulses per point (PPP). Treatment points were spaced 3.5 mm axially and 1.5 mm laterally and vertically to ensure adequate overlap of the cavitation bubble cloud. Acoustic output gradually increased until consistent bubble cloud formation was observed within the target lesions. Although direct measurements of in situ focal pressure were not feasible, the estimated peak negative pressure at the focus was calculated assuming 4 cm of overlying tissue and an attenuation coefficient of 0.5 dB/cm/MHz. Across the study, the estimated in situ peak negative pressure (PNP) was 22.73 ± 0.57 MPa. In one pig, the estimated in situ pressures were variable during treatment and ranged from 15.24 to 25.94 MPa. Each tumor underwent two complete volumetric treatments using the described parameters. Immediately following histotripsy treatment, one pig from each round was evaluated with CT imaging to assess treatment effects.

### 2.5. Necropsy and Histopathology

Immediately following euthanasia, gross evaluation of all animals was performed by a board-certified veterinary pathologist (S.C.-O. or K.E.). Following comprehensive examination of the abdomen and viscera for abnormalities, the liver was removed en bloc and serially sectioned. Tissue samples were taken from grossly identifiable tumors (treated and untreated), metastases, and any other identifiable abdominal abnormalities. Additionally, when deemed relevant, samples were collected from the proximal and distal portions of the ablation zone. All harvested tissues were fixed in formalin, embedded in paraffin, and stained with hematoxylin and eosin (H&E).

### 2.6. Blood Chemistry Analysis

Blood samples were collected from the jugular veins before and after histotripsy treatment to measure biochemical markers that assess liver function. Samples were collected using non-additive BD Vacutainer tubes, and serum was isolated by centrifugation. Serum samples were submitted to the diagnostic lab at Virginia Tech Animal Laboratory Services (VITALS) for measurement of Alanine aminotransferase (ALT), Alkaline phosphatase (ALKP), direct bilirubin, indirect bilirubin, total bilirubin, and Creatinine (CK) levels.

## 3. Results

### 3.1. Successful Engraftment of Human Pancreatic and Hepatocellular Carcinoma Cells in the Porcine Liver

Ten RAG2/IL2RG immunocompromised pigs were used to establish human tumor xenografts in the liver. Two separate studies were conducted, with four pigs in the first study and six in the second study. To generate immunocompromised pigs, 1265 in vitro oocytes underwent CRISPR/Cas9 editing targeting the porcine RAG2 and IL2RG genes before culture [18]. Genomic DNA from ear notch samples confirmed successful gene targeting in all 10 piglets. Representative genotypes are shown in Supplementary Figure S1.

Panc-1 and HepG2/C3A cells were engrafted into the liver parenchyma to establish orthotopic tumors. Each pig received two tumor engraftments. HepG2/C3A cells were injected into the right medial liver lobe, and Panc-1 cells were injected into the left medial liver lobe. Successful tumor engraftment was achieved across the study cohort, enabling subsequent evaluation of histotripsy treatment. One control animal with engrafted tumors that did not receive histotripsy treatment was included in each study to determine baseline conditions, tumor progression, and procedural effects and for imaging optimization.

### 3.2. Tumors Confirmed Using CT Imaging Prior to Histotripsy Treatment

Computed tomography (CT) was performed before histotripsy treatment to confirm tumor engraftment, location, and size. One pig was selected from each study for pre-treatment imaging to assess tumor establishment and to facilitate treatment planning. Contrast-enhanced CT scans revealed distinct focal lesions within the right medial lobe of the liver, consistent with the successful tumor engraftment, Pre-ablation Hounsfield units: Pre contrast: 75.25 HU, post contrast: 85 HU (Figure 1). On the CT image, the lesion appeared as a well-defined hypodense region relative to the surrounding hepatic parenchyma, indicating localized tumor growth within the liver (Figure 1). The lesion margins were clearly distinguishable from adjacent liver structures, confirming the tumor’s precise location for treatment planning. These pre-treatment CT images were also used to evaluate the anatomy and the relationship between the tumor and the surrounding hepatic structures, which is particularly important for planning histotripsy targeting in regions considered difficult to access. Representative images demonstrate the hepatic lesion in the right medial lobe next to the gallbladder (Figure 1). The tumor region is highlighted with arrows, showing the contrast between the hypodense tumor and the surrounding normal liver tissue (Figure 1). Overall, pre-treatment CT imaging provided critical confirmation of tumor localization and served as a crucial step in guiding subsequent histotripsy planning and treatments.

### 3.3. Successful Histotripsy Treatment of Liver Tumors Confirmed by CT Imaging

**Figure 3:**
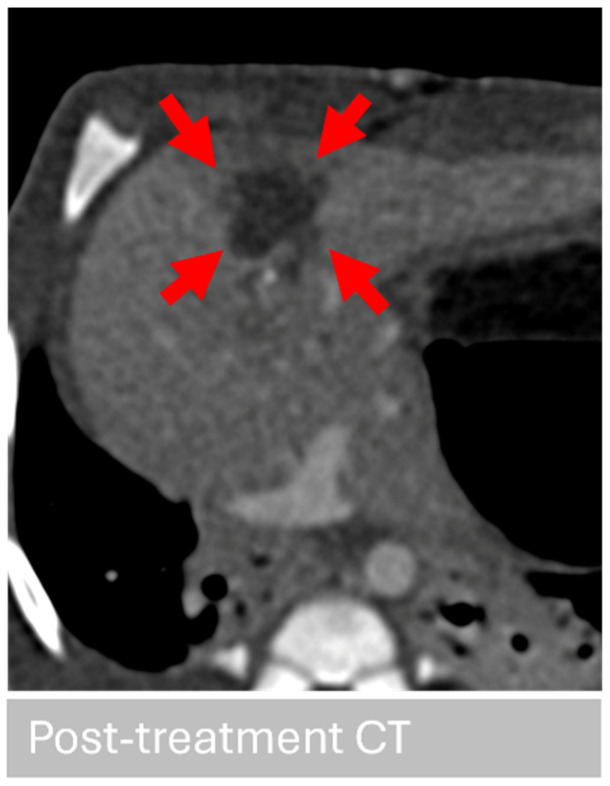
Representative post-treatment CT image indicating successful ablation of tumor. Red arrows define treatment zone post-histotripsy with complete coverage of the tumor by the non-enhancing treatment zone. The ablation zone size was elongated, 19.29 × 11.53 × 11.77 mm (L × W × H). Post-ablation Hounsfield Units: Pre contrast: 57.68 HU, post contrast: 57.98 HU.

Histotripsy was successfully delivered to targeted tumor sites in all treated animals with acoustic targeting guided by intraoperative ultrasound imaging (Figure 2). The planned treatment grid and the 5 mm margin were achieved according to the protocol parameters. CT imaging was performed after histotripsy treatment to assess treatment accuracy and validate tumor ablation success (Figure 3). CT assessments showed clear changes at the targeted sites within the liver, consistent with tissue disruption (Figure 3). In treated animals, the ablation zones were identified as regions of altered tissue density relative to surrounding hepatic tissue (Figure 3). These regions matched the treatment locations previously identified on pre-treatment imaging and intraoperative ultrasound guidance. The ablation zones were well localized to the targeted tumor, demonstrating the spatial precision of the treatment. Comparing pre- and post-treatment images confirmed the effective targeting and ablation of the hepatic tumor. Representative CT images of the ablation zones are shown, with treated regions marked by arrows in Figure 3. We also performed quantitative measurements of the ablation zones on CT (long axis, short axis, and craniocaudal) to compare with the planned treatment volume. For the representative CT images shown in Figure 3, the ablation zone size was elongated, 19.29 mm × 11.53 mm × 11.77 mm (L × W × H). Post-ablation Hounsfield Units: Pre contrast: 57.68 HU, post contrast: 57.98 HU (Figure 3). Quantitative analysis indicated a reduction in attenuation and loss of contrast enhancement after histotripsy treatment, which is consistent with histotripsy-treated tissue [46] (verified by a board-certified veterinary radiologist (M.E. and T.Z). Together, the CT findings were useful in providing radiographic confirmation of successful histotripsy treatment within the liver of the immunocompromised pigs.

### 3.4. Necropsy Confirms Tumor Engraftment and Successful Ablation

**Figure 4:**
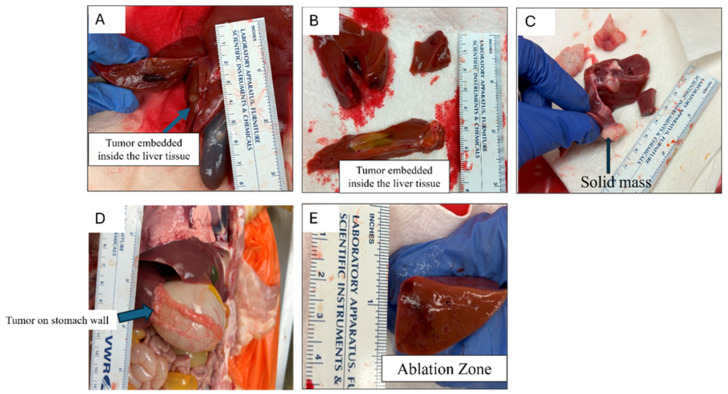
Macroscopic confirmation of tumor engraftment, metastatic tumor development, and successful histotripsy ablation. (**A**–**C**) Representative gross images demonstrating successful engraftment of tumors within the liver parenchyma. Tumors appear as well-defined lesions embedded in hepatic tissue. (**D**) In some animals, small lesions were identified on distal organs, including the stomach wall. (**E**) Representative gross image of the treatment site following histotripsy, showing a clearly demarcated ablation zone within the liver tissue, indicative of effective mechanical tissue fractionation.

Following the completion of each study, necropsies were performed on all animals to evaluate tumor engraftment, metastasis, and ablation effects. Gross confirmation of tumor engraftment was identified in 8/10 pigs. Grossly, tumors manifested as 0.4–1.0 cm, soft, pale tan to white masses visible along the capsular surface and consistently extending into the underlying liver tissue. These were identified at one or more sites in each animal, including the targeted injection sites of the right and left medial liver lobes. Additionally, multiple animals exhibited evidence of one or more similar masses at distant sites within the liver (intrahepatic metastasis), and/or implantation along one or more abdominal surfaces (carcinomatosis). Gross lesions consistent with histotripsy treatment were identified in all animals treated with histotripsy. These were characterized by localized but variably sized foci of hemorrhaging that were often abruptly demarcated from the surrounding tissue, with some animals also exhibiting formation of large blood clots at the surface of the treated region (Figure 4A–E). These gross findings were consistent with mechanical tissue fractionation caused by histotripsy and were specifically aligned with the tumor locations identified during imaging and treatment planning. Additionally, two of three treated animals in the second study did show gross evidence of off-target effects. These included hemorrhage along the outer surface of the adjacent stomach wall in one animal as well as hemorrhage along an adjacent region of gallbladder in another. Untreated control animals, which had tumors engrafted but had no histotripsy treatment, exhibited no evidence of hemorrhage or tissue damage as identified in histotripsy treated animals.

### 3.5. Histological Confirmation of Tumor Engraftment and Histotripsy-Mediated Ablation

**Figure 5:**
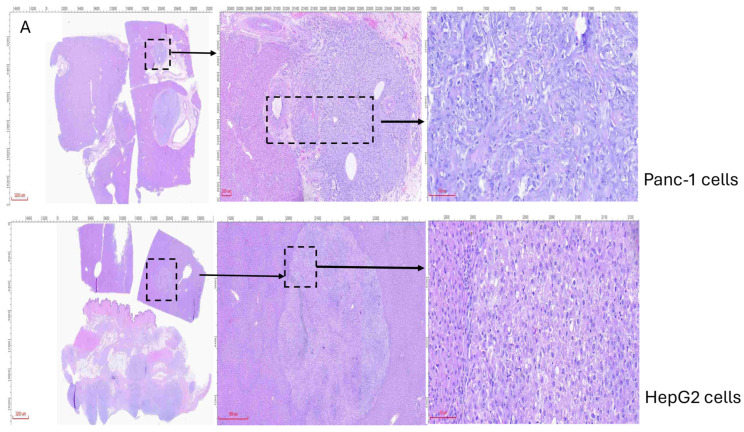

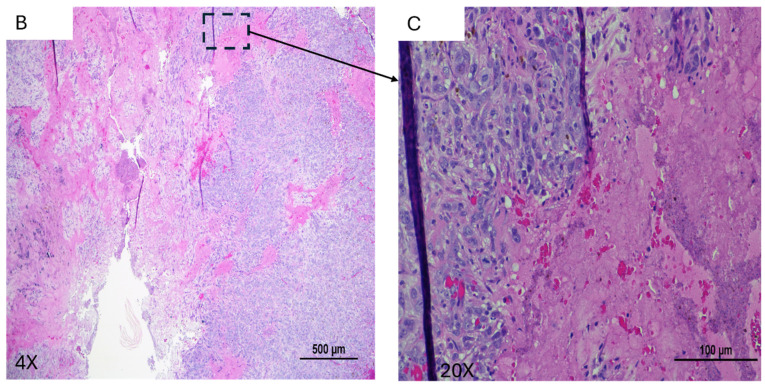
Histological characterization of histotripsy ablation of orthotopic Panc-1 and HepG2/CEA tumors in the porcine liver model. (**A**–**C**) Representative hematoxylin and eosin (H&E) stained sections of liver tissue demonstrating the presence of two tumor morphologies. (**A**) Regions interpreted as pancreatic adenocarcinoma (Panc-1) display more irregular, densely packed cells with disorganized architecture and more stromal background. In contrast, those interpreted as HepG2/C3A exhibit polygonal cells with centrally located nuclei and trabecular growth patterns. (**B**) Sections of the tumor tissue following histotripsy treatment (4× magnification) demonstrate well-defined ablation zones characterized by disruption of normal tissue architecture. (**C**) Treated regions appear pink in color (20× magnification), showing marked evidence of mechanical tissue disruption and acellular debris consistent with histotripsy-induced ablation, with clear margins between treated and untreated areas of the tumor.

Histopathology was performed on liver and other relevant tissues collected from all animals to further assess tumor engraftment, metastasis, and ablation effects. Successful engraftment of tumor cells was confirmed histologically with grossly identifiable masses and exhibited two general morphologies (Figure 5A). The most commonly identified tumors were composed of highly pleomorphic (abnormal) round to polygonal to elongate cells arranged in nests, packets, and vague streams often associated with robust desmoplasia (abnormal fibrosis induced by malignant epithelial cells), supportive of Pan02 tumors (Figure 5A). These were frequently identified at sites of injection but also composing intrahepatic and abdominal metastases. These cells were also frequently identified in vessels. The second population of tumor cells was less consistently identified and was composed of cohesive clusters and trabecular arrangements of polygonal cells with hepatocyte-like cellular morphology. These were not associated with a scirrhous response but rather vaguely recapitulated hepatic cords separated by a fine stroma reminiscent of hepatic sinuses (Figure 5A). This latter population was interpreted as HepG2 tumors, which were only occasionally identified as composing intrahepatic metastases.

Histopathology of grossly confirmed, histotripsy-treated regions revealed clear evidence of mechanical tissue disruption, consistent with successful tissue targeting and ablation. Treated areas were characterized by varying degrees of tissue disintegration and hemorrhage within the ablation zone as well as sporadic lesions consistent with acute vascular damage. Disintegrated tissue was characterized by complete loss of preexisting architecture and replacement by acellular homogenate containing fine nuclear debris consistent with cavitation and mechanical destruction caused by the treatment (Figure 5B,C). Vascular damage was characterized by varying degrees of vessel wall disruption as well as expansion by fibrin. The stomach and gallbladder from the two animals that exhibited gross evidence of off-target effects in these tissues were also examined histologically. A localized region of hemorrhage, edema, and vascular damage was confirmed on the surface of the stomach of the former, and a small perforation in the wall of the gallbladder was confirmed in the latter.

Together, these histological findings confirmed successful orthotopic engraftment of both human pancreatic and hepatocellular tumors within the immunocompromised pig liver and provide direct pathological evidence of effective tumor ablation following histotripsy treatment (Figure 5A–C).

### 3.6. Serum Biochemistry Indicates Physiological Response to Tumor Growth and Treatment

**Figure 6:**
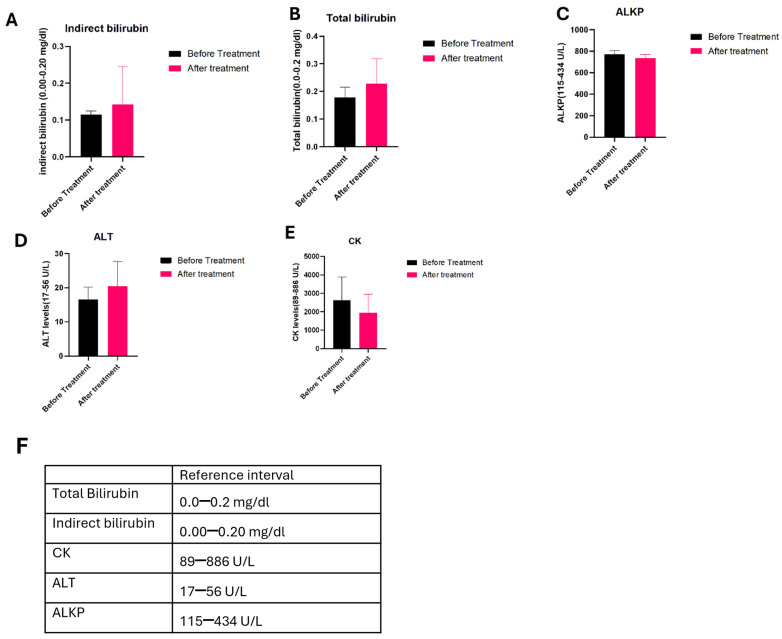
No significant differences in serum liver function biomarkers following histotripsy treatment. (**A**) Indirect and (**B**) total bilirubin levels before and after histotripsy treatment. (**C**) Alkaline phosphatase (ALKP) and (**D**) Alanine aminotransferase (ALT) levels before and after treatment. (**E**) CK levels before and after treatment. (**F**) Normal reference intervals for each marker. *n* = 4 pigs for each biomarker.

Serum chemistry profiles were analyzed to monitor systemic responses during tumor establishment and histotripsy treatment. Following tumor cell implantation but prior to histotripsy treatment, several biochemical markers associated with tissue injury and hepatic function, including creatine kinase (CK), alanine aminotransferase (ALT), alkaline phosphates (ALKP), and bilirubin, were elevated in all tumor-bearing pigs relative to established reference intervals (Figure 6). Despite these elevations relative to baseline reference ranges, no significant increases beyond this were identified in animals following histotripsy treatment (Figure 6). These findings suggest that the observed biochemical changes are primarily attributable to tumor burden and associated hepatic stress rather than histotripsy treatment, supporting the safety and tolerability of the treatment in this model (Figure 6).

## 4. Discussion

In this study, we developed and evaluated a large-animal platform for focused ultrasound-based liver tumor ablation using orthotopically engrafted Panc-1 and HepG2/C3A tumors. By leveraging CRISPR/Cas9-generated immunocompromised pig models [18], we achieved a highly reliable tumor engraftment, demonstrating the model’s robustness. The presence of tumors was confirmed through multiple complementary approaches, including imaging, gross necropsy, and histological analysis. Together, these findings demonstrate the utility of the immunocompromised pig model provides a robust and clinically relevant platform for studying liver and tumor biology and evaluating emerging local/regional therapies.

A key outcome of this work is the successful establishment of human tumor xenografts in the liver of a large-animal model. Many pre-clinical investigations rely on small-animal models that cannot adequately replicate the anatomy, microscale, vascular architecture, and imaging environment encountered in human liver cancer treatment. The use of immunocompromised pigs provides a permissive host environment that supports human tumor development while maintaining physiological characteristics that closely resemble those of human patients [18,19,20,21]. The consistency of tumor engraftment observed across animals further highlights the biological robustness of the model system. Imaging played an important role in confirming tumor establishment and guiding treatment planning in this study. Pre-treatment CT images revealed a clearly defined hypodense lesion corresponding to the tumor implantation site, enabling accurate localization of the tumors within the liver for targeting and subsequent histopathology. The use of clinically relevant imaging modalities is a significant advantage of large-animal models, as it enables a therapeutic workflow that closely mirrors that used in interventional oncology. Importantly, comparison of pre- and post-treatment CT scans demonstrated distinct changes in tissue density at treated sites, providing radiographic evidence of successful tumor ablation, further highlighting the model’s translational potential. Beyond the evaluation of histotripsy, the model described here may provide a versatile platform for studying a range of therapeutic strategies targeting liver malignancies. Because tumors can be orthotopically engrafted within specific hepatic regions, this system may enable future studies investigating tumor growth dynamics, treatment responses in challenging anatomical locations, and combination strategies integrating locoregional and systemic therapies. Additionally, the ability to engraft human tumor cells within a clinically relevant anatomical context offers opportunities to investigate tumor microenvironment interactions and therapeutic responses that may be difficult to replicate in smaller animal models [45,47,48].

This work expands upon our prior studies, engrafting Panc-1 tumors in the pancreas of these immunocompromised pigs [18]. The study successfully engrafted orthotopic Panc-1 tumors in immunocompromised pigs, ablated the tumors, and demonstrated the reduction of collagenous stroma in the tumors after histotripsy treatment. Increased metastasis was a notable difference between our prior studies in the pancreas and the current studies in the liver [18]. In the current liver study, two animals exhibited suspected “too numerous to count” (TNTCs) tumor lesions. In these animals, a greater proportion of these tumors were suspected to be derived from the Panc-1 cells. This observation may reflect the aggressive, proliferative, and invasive behavior of pancreatic cancer cells within the hepatic microenvironment. While this study was not designed to compare tumor growth kinetics between the two cell types directly, these findings are consistent with the well-recognized aggressiveness of pancreatic ductal adenocarcinoma [49,50,51]. Clinically, the liver is the most common site of metastasis for pancreatic cancer, and the predominance of pancreatic tumor growth observed in the liver model may therefore reflect the strong biological tropism of pancreatic cancer cells for hepatic tissue. While there have been relatively few studies to evaluate multiple tumor types within the same hepatic model, previous studies have demonstrated that metastatic organotropism mostly depends on tumor type, with distinct cancel cell populations demonstrating differential capacity for liver colonization [52]. However, because imaging and histological confirmation of these TNTCs were not performed, the origin of these lesions could not be definitively established. These findings may reflect injection-site leakage rather than true metastatic spread and should therefore be interpreted with caution.

Beyond the novelty of the model, we were also able to demonstrate effective histotripsy ablation of both hepatocellular carcinoma and pancreatic adenocarcinoma tumors in the liver in the current study. This was observed and confirmed through multiple lines of evaluation. Gross necropsy revealed a clearly demarcated ablation zone corresponding to the planned treatment location and size, while histopathology examination provided further confirmation of treatment effects. In sum, our data show significant mechanical tissue disruption in the targeted tumors, consistent with histotripsy ablation. Treated areas were characterized by loss of normal hepatic architecture, cellular debris, hemorrhage, and necrotic hepatocytes. The sharply defined ablation zones observed in the study aligned with previous reports demonstrating that histotripsy can produce precise mechanical ablation while limiting collateral damage to surrounding tissue. Likewise, we did not observe any significant differences in biomarkers associated with liver damage or injury following histotripsy treatment. As this was a preliminary acute proof-of-concept study, immune responses and long-term tissue remodeling were not evaluated [53,54].

Together, these findings further support the safety and efficacy of histotripsy as a therapeutic modality in the liver. These findings are consistent with observations from ongoing human clinical trials [39,40]. Initial first in-human and multicenter studies including THERESA and HOPE4LIVER have demonstrated high rates of immediate success with a favorable short-term safety profile. Initial findings from the THERESA feasibility study demonstrated a median tumor volume reduction of 40–70% within 3 months following treatment. These early outcomes were further supported by the multicenter HOPE4LIVER trial, which reported a 90% local tumor control rate at 1 year. Additionally, one-year overall survival rates were 73.3% for patients with primary liver malignancies and 48.6% for those with metastatic liver lesions, highlighting the promising clinical potential of histotripsy for the treatment of hepatic tumors. Notably, histotripsy produced sharply defined treatment zones and remained effective in proximity to major vascular structures.

Despite these advantages, important challenges remain, including studies evaluating improved targeting approaches and optimal treatment parameters for different tumor types or characteristics. In this context, the present study provides a clinically relevant large-animal platform to further investigate histotripsy in heterogeneous liver disease. This model will help to address the current clinical limitations and guide optimization of treatment strategies.

Overall, this work demonstrates the successful establishment of orthotopic human pancreatic and hepatocellular tumors in an immunocompromised pig model and provides multimodal confirmation of histotripsy-mediated tumor ablation. Imaging, gross pathology, and histological analysis collectively demonstrated precise, mechanical tumor destruction at the targeted treatment site by integrating a clinically relevant tumor model with focused ultrasound-based therapy. Histotripsy was confirmed to be safe and effective for use in treating liver tumors. This work provides a valuable translational framework for advancing histotripsy as a treatment strategy for primary liver malignancies and metastatic pancreatic malignancies and for future limitation testing of this tumor ablation modality.

## 5. Conclusions

In conclusion, this study describes the successful development of a clinically relevant orthotopic porcine model of both primary and metastatic liver cancer through the engraftment of human hepatocellular carcinoma and pancreatic adenocarcinoma cells in immunocompromised pigs. Using this platform, we demonstrated the safety and feasibility of histotripsy as a non-invasive, non-thermal ablation modality for liver tumors. Real-time treatment visualization, combined with imaging, gross necropsy, and histological analyses, confirmed successful engraftment of tumors, accurate targeting, and effective destruction of tumor tissue. Furthermore, the absence of significant changes in serum biomarkers following histotripsy treatment supports the favorable safety profile of histotripsy and its ability to ablate targeted tissue while preserving overall liver function.

These findings establish a valuable large-animal translational model for the evaluation and optimization of histotripsy in liver malignancies. Therefore, the model addressed a critical gap between small animal studies and clinical application by providing a physiologically and anatomically relevant model for investigating histotripsy treatment strategies and therapeutic outcomes. Further studies using this model will facilitate the refinement of histotripsy protocols, investigation of tumor-specific responses, and evaluation of combination therapeutic strategies, thereby supporting the continued advancement and broader application of histotripsy in the management of primary and metastatic liver cancers.

## Data Availability

The original contributions presented in this study are included in the article/Supplementary Materials. Further inquiries can be directed to the corresponding authors.

## Supplementary Materials

The following supporting information can be downloaded at: https://www.mdpi.com/article/10.3390/cancers18152432/s1, Figure S1: All pigs were genotyped to confirm successful genetic modification of the RAG2 and IL2RG gene. A representative image showing sequencing chromatograms that indicate the specific indels generated by the CRISPR/Cas9 system. The target sites for sgRNAs are highlighted in light blue. Figure S2: Histotripsy Therapy System. (A) Clinical histotripsy cart and coaxial US imaging probe mounted onto a micropositioner. (B) Zoomed-in version of the UMC bowl with therapy transducer submerged via an adjustable arm.
