# Supplementary material for: Safe and Effective Histotripsy Ablation of Human Liver Tumors in a Genetically Modified Porcine Model
Source: Cancers (Basel). 2026 Jul 29;18(15):2432. doi: 10.3390/cancers18152432 (PMC13464800; doi:10.3390/cancers18152432)
Supplement: Supplementary file 1 [file cancers-18-02432-s001.zip › cancers-4440927-supplementary.pdf]

cancers

Supplementary Material: Safe and Effective Histotripsy Ablation of Human Liver Tumors in a Genetically Modified Porcine Model

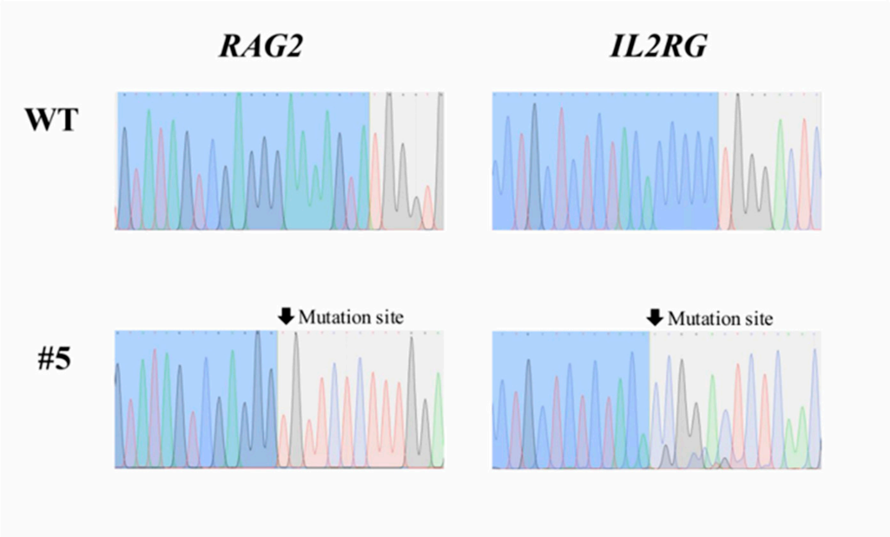

Figure S1. All pigs were genotyped to confirm successful genetic modification of the RAG2 and IL2RG gene. A representative image showing sequencing chromatograms that indicate the specific indels generated by the CRISPR/Cas9 system. The target sites for sgRNAs are highlighted in light blue.

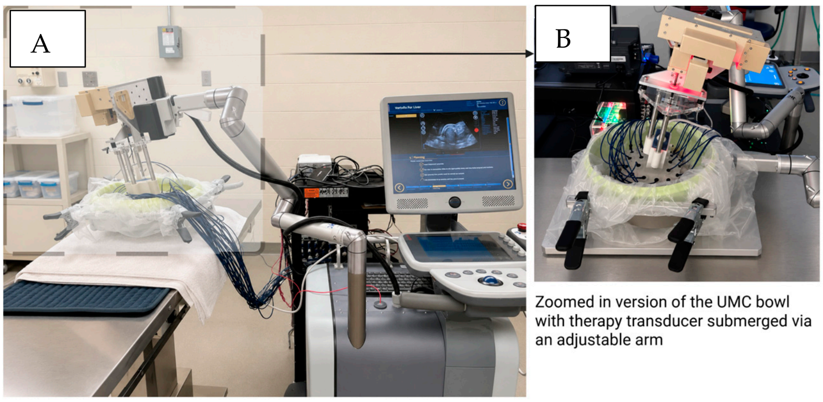

Figure S2. Histotripsy Therapy System. (A) Clinical histotripsy cart and coaxial US imaging probe mounted onto a micropositioner. (B) Zoomed-in version of the UMC bowl with therapy transducer submerged via an adjustable arm.

 https://doi.org/10.3390/cancers18152432
